# 18F-Fluorodeoxyglucose Positron Emission Tomography Tracks the Heterogeneous Brain Susceptibility to the Hyperglycemia-Related Redox Stress

**DOI:** 10.3390/ijms21218154

**Published:** 2020-10-31

**Authors:** Alberto Miceli, Vanessa Cossu, Cecilia Marini, Patrizia Castellani, Stefano Raffa, Maria Isabella Donegani, Silvia Bruno, Silvia Ravera, Laura Emionite, Anna Maria Orengo, Federica Grillo, Flavio Nobili, Silvia Morbelli, Antonio Uccelli, Gianmario Sambuceti, Matteo Bauckneht

**Affiliations:** 1 Department of Health Sciences, University of Genoa, 16132 Genova, Italy; albertomiceli23@gmail.com (A.M.); vane.6291@gmail.com (V.C.); Stefanoraffa@live.com (S.R.); isabella.donegani@gmail.com (M.I.D.); silviadaniela.morbelli@hsanmartino.it (S.M.); sambuceti@unige.it (G.S.); 2 Nuclear Medicine, IRCCS Ospedale Policlinico San Martino, 16132 Genova, Italy; cecilia.marini@unige.it (C.M.); annamaria.orengo@hsanmartino.it (A.M.O.); 3 CNR Institute of Molecular Bioimaging and Physiology (IBFM), 20090 Milano, Italy; 4 Cell Biology, IRCCS Ospedale Policlinico San Martino, 16132 Genova, Italy; patrizia.castellani@hsanmartino.it; 5 Department of Experimental Medicine, Human Anatomy, University of Genoa, Genova 16132, Italy; silvia.bruno@unige.it (S.B.); silvia.ravera@unige.it (S.R.); 6 Animal Facility, IRCCS Ospedale Policlinico San Martino, 16132 Genova, Italy; laura.emionite@hsanmartino.it; 7 Department of Surgical Sciences and Integrated Diagnostics, Pathology Unit, University of Genoa, 16132 Genova, Italy; federica.grillo@unige.it; 8 Department of Neuroscience, Rehabilitation, Ophthalmology, Genetics, Maternal and Child Health, Center of Excellence for Biomedical Research, University of Genoa, 16132 Genoa, Italy; flaviomariano.nobili@hsanmartino.it (F.N.); auccelli@neurologia.unige.it (A.U.); 9 Clinical Neurology, IRCCS Ospedale Policlinico San Martino, 16132 Genoa, Italy

**Keywords:** brain glucose metabolism, FDG-PET, hyperglycemia, oxidative stress, reticular pentose phosphate pathway

## Abstract

In cognitively normal patients, mild hyperglycemia selectively decreases 18F-Fluorodeoxyglucose (FDG) uptake in the posterior brain, reproducing Alzheimer disease pattern, hampering the diagnostic accuracy of this widely used tool. This phenomenon might involve either a heterogeneous response of glucose metabolism or a different sensitivity to hyperglycemia-related redox stress. Indeed, previous studies reported a close link between FDG uptake and activation of a specific pentose phosphate pathway (PPP), triggered by hexose-6P-dehydrogenase (H6PD) and contributing to fuel NADPH-dependent antioxidant responses in the endoplasmic reticulum (ER). To clarify this issue, dynamic positron emission tomography was performed in 40 BALB/c mice four weeks after administration of saline (*n* = 17) or 150 mg/kg streptozotocin (*n* = 23, STZ). Imaging data were compared with biochemical and histological indexes of glucose metabolism and redox balance. Cortical FDG uptake was homogeneous in controls, while it was selectively decreased in the posterior brain of STZ mice. This difference was independent of the activity of enzymes regulating glycolysis and cytosolic PPP, while it was paralleled by a decreased H6PD catalytic function and enhanced indexes of oxidative damage. Thus, the relative decrease in FDG uptake of the posterior brain reflects a lower activation of ER-PPP in response to hyperglycemia-related redox stress in these areas.

## 1. Introduction

Glucose is the main metabolite used by the brain to fuel neuronal activity, neurotransmitter reuptake, and maintenance of neuronal membrane polarization [1]. Providing a precise assessment of the local cerebral metabolic rate of glucose consumption, 18F-Fluorodeoxyglucose positron emission tomography (FDG PET) is widely used, in the clinical setting, to non-invasively map alterations in neuronal density and synapse dysfunction to facilitate the early and differential diagnosis of mild cognitive impairment and dementia [2]. Technical considerations prevent the application of this technique in severely hyperglycemic patients. In fact, the high competition by unlabeled glucose for transmembrane transporters decreases brain FDG uptake, thus enhancing stochastic noise and lowering overall image quality [3]. Accordingly, the European Association of Nuclear Medicine (EANM) guidelines currently contraindicate brain FDG imaging in patients with serum glucose levels >160 mg/dL [4]. 

However, several clinical studies showed that even mild hyperglycemia hampers tracer retention, leading to uptake defects in the precuneus, posterior cingulate, parietal, and occipital brain regions [5,6,7,8] regardless of the presence of cognitive impairment. On the clinical ground, this inhomogeneity mimics the radioactivity distribution pattern usually observed in Alzheimer disease (AD). From the pathophysiological point of view, the heterogeneous brain response to the same increase in glucose availability indicates that regulation of sugar metabolism might be different in anterior and posterior cortical regions. In other words, it hampers the current assumption linking FDG uptake, glucose consumption, and synaptic activity. 

FDG radioactivity is considered a robust index of overall tissue glucose consumption based on the notion that once entered the cytosol, this glucose analog is phosphorylated by hexokinases (HK) to FDG-6P that is irreversibly trapped as a terminal metabolite [9]. However, magnetic resonance spectroscopy documented a significant degradation of FDG-6P [10,11,12,13]. Furthermore, FDG uptake has been found to be relatively independent of glycolytic flux and strictly connected to the activity of hexose-6-phosphate dehydrogenase (H6PD): An enzyme confined within the endoplasmic reticulum (ER) and able to catalyze a wide variety of phosphorylated hexoses (including FDG-6P), triggering a local pentose phosphate pathway (PPP) [13,14,15,16,17,18,19,20,21]. In mammalian tissues, PPP is dedicated mainly to reducing NADP equivalents to NADPH to fuel either bio-reductive syntheses or NADPH-dependent antioxidant responses. Consequently, the inhomogeneous brain FDG uptake observed under moderate hyperglycemic conditions might reflect a heterogeneous regulation of glucose metabolism as well as a heterogeneous vulnerability to the redox stress among the various brain regions. 

On these bases, the present study aimed to verify the link between brain FDG uptake, glucose metabolism, ER-PPP activation, and redox stress in anterior and posterior cortical areas in an experimental model of sustained moderate hyperglycemia.

## 2. Results

### 2.1. Effect of STZ on Serum Glucose Levels and Brain FDG Kinetics

**Figure 1:**
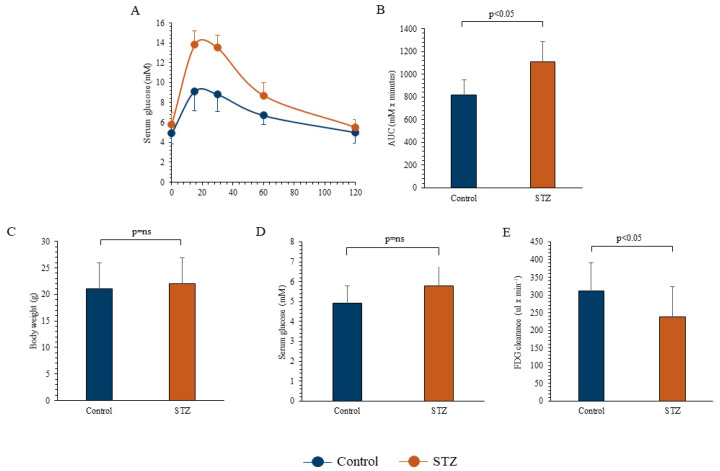
Effect of streptozotocin (STZ) on mice serum glucose level, body weight, and clearance. Serum glucose levels at oral glucose tolerance test (OGTT) (Panel **A**) and area under the curve (Panel **B**) in the 17 control (blue) and in the 23 STZ-treated mice (brown). Body weight (Panel **C**) and average fasting glycemia (Panel **D**) were measured two weeks after OGTT, soon before PET imaging, in control (*n* = 17, blue column) and STZ groups (*n* = 23, brown column). Blood 18F-Fluorodeoxyglucose (FDG) clearance was significantly decreased in STZ mice (Panel **E**). Data are expressed as mean ± SD.

The study protocol included 40 6-week-old male BALB/c mice. A subgroup (*n* = 23) received a single intraperitoneal administration of Streptozotocin (STZ) at the dose of 150 mg/kg, while the remaining 17 controls received saline. The study was completed in all mice, and no visible side effects occurred after STZ administration. STZ effect was confirmed by an oral glucose tolerance test (OGTT) that was performed two weeks after drug administration and documented a higher area under the curve of serum glucose level with respect to controls (Figure 1A,B). Two weeks thereafter, mice underwent FDG micro-PET imaging. At the time of imaging, mice body weight was similar in both groups (Figure 1C) and fasting serum glucose level was only slightly, though not significantly, higher in STZ with respect to controls (Figure 1D). However, STZ mice showed a significant decrease in blood FDG clearance (Figure 1E).

**Figure 2:**
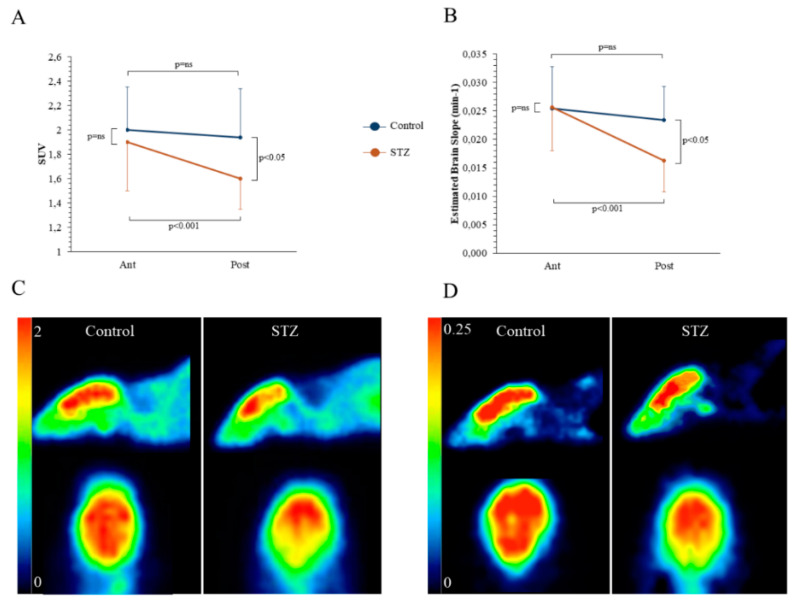
In vivo effects of hyperglycemia on Standardized Uptake Value (SUV) and slope of Patlak. Average SUV (Panel **A**) and slope of Patlak regression line (*k*_1_ × *k*_3_)/(*k*_2_ + *k*_3_) (Panel **B**) of anterior (Ant) and posterior (Post) brain areas in control (*n* = 17, blue) and STZ mice (*n* = 23, brown). Hyperglycemia significantly decreased both parameters in posterior brain regions. Representative images of axial and sagittal projection representing the average SUV (Panel **C**) and the parametric reconstruction of the Patlak slope (Panel **D**).

At conventional analysis, brain FDG uptake was homogeneous in control mice. In fact, posterior and anterior standardized uptake values (POST-SUV and ANT-SUV, respectively) were superimposable (Figure 2A). By contrast, STZ selectively decreased FDG uptake in posterior cortical regions, in which SUV was lower with respect to both the corresponding anterior brain areas and the posterior segments of controls (Figure 2A). This phenomenon was coherent with the behavior of the avidity for the tracer defined by the slope (*k*_1_ × *k*_3_)/(*k*_2_ + *k*_3_) of Patlak regression line that was selectively reduced by chronic hyperglycemia only in the posterior brain of STZ mice (Figure 2B). Emblematic examples of images from control and STZ mice are reported in Figure 2C,D, for SUV and slope regression line, respectively. Serum glucose level was not correlated with the posterior/anterior ratio of either SUV or regression line slope (data not shown).

### 2.2. Evaluation of Enzymatic Catalytic Functions 

**Figure 3:**
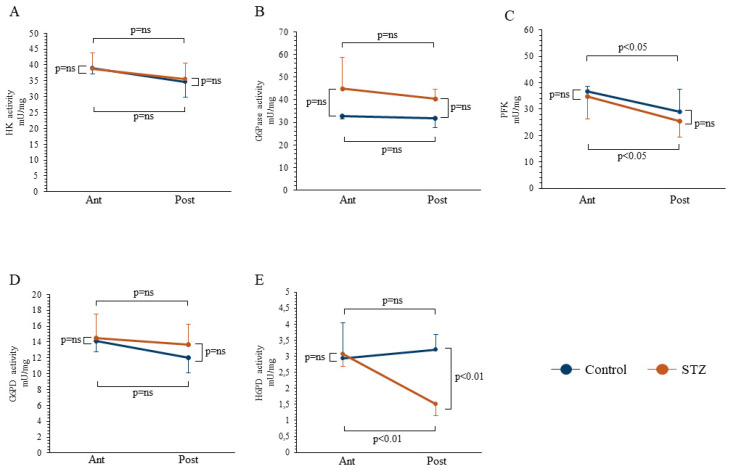
Effect of STZ treatment on enzymatic pathway of glucose degradation. Catalytic activities of hexose-kinase (HK, Panel **A**), glucose-6-phosphatase (G6Pase, Panel **B**), Phosphofructokinase (PFK, Panel **C**), glucose-6-phosphate dehydrogenase (G6PD, Panel **D**), and hexose-6-phosphate dehydrogenase (H6PD, Panel **E**) in anterior (Ant) and posterior (Post) brain areas in control (*n* = 6 for each area, blue) and STZ mice (*n* = 6 for each area, brown). STZ treatment significantly reduced H6PD catalytic function in posterior brain areas, while it did not affect the main enzymes involved in glucose entrapment, release, and cytosolic catabolism. Data are expressed as mean ± SD.

Spectrophotometric analysis showed that the heterogeneity in FDG distribution was independent of the activity of the main enzymes responsible for glucose metabolism. Indeed, HK catalytic function was remarkably similar in the anterior and posterior brain regions, irrespective of the presence or absence of chronic hyperglycemia (Figure 3A). On the other hand, G6Pase function was slightly (though not significantly) increased by STZ. However, this effect similarly involved both the anterior and posterior brain areas (Figure 3B). Similarly, the behavior of the triggering enzyme of glycolysis, Phosphofructokinase (PFK), was not adherent to FDG retention: Enzyme activity was actually lower in posterior than in anterior brain regions; however, this difference was reproduced both in control and STZ mice (Figure 3C). We, thus, moved our attention to the cytosolic PPP: G6PD catalytic function, which was similar in both anterior and posterior regions of STZ and control mice (Figure 3D). Therefore, the analysis of the major regulators of the cytosolic glucose metabolism did not explain the observed regional effect of hyperglycemia on brain FDG uptake.

By contrast, the response of FDG uptake to previous STZ administration agreed with the behavior of H6PD enzymatic activity. While the catalytic function was superimposable in the anterior cerebral regions harvested from either group (Figure 3E), it was selectively decreased in the posterior brain of STZ mice to values significantly lower compared to both the corresponding anterior segments and the posterior brain of controls, indicating a selective response, as also confirmed by the synergism analysis (*p* = 0.03). The enzymatic activity data were confirmed by the Western Blot (WB) analyses, which showed that the differences in enzymatic activities were paralleled by changes in enzymatic expression (Supplementary Figure S1).

### 2.3. Regional Heterogeneity of the Hyperglycemia-Related Redox Damage

**Figure 4:**
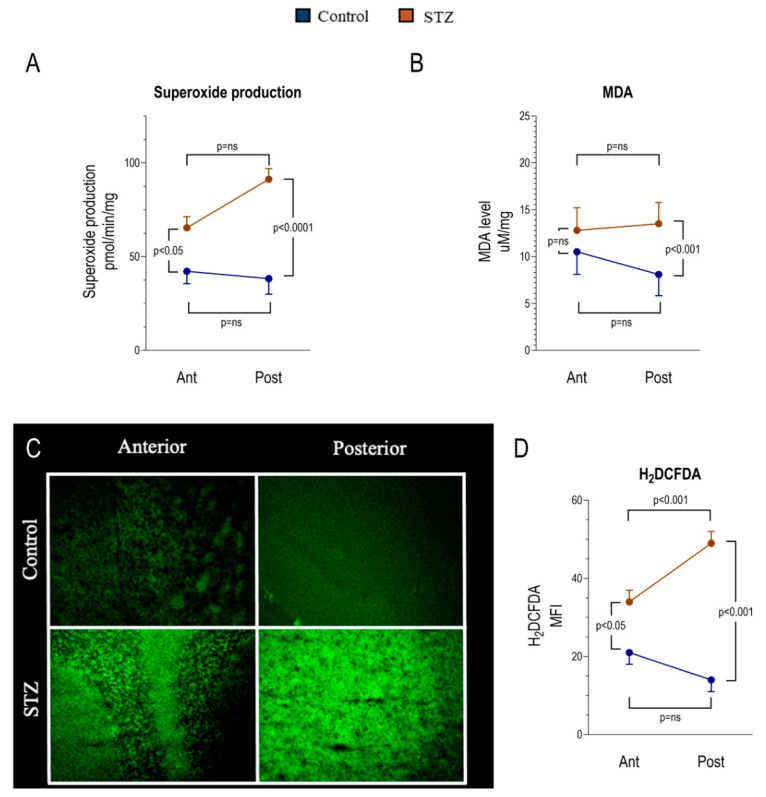
Hyperglycemia effect on oxidative state of anterior and posterior brain areas as assessed by spectrophotometry and cellular fluorescence. Panels **A** and **B** show the degree of superoxide production and malondialdehyde (MDA) content in the anterior (Ant) and posterior (Post) brain regions in control (*n* = 6 for each area, blue) and STZ mice (*n* = 6 for each area, brown). Panels **C** and **D** display representative images of anterior and posterior brain areas stained with 2′,7′-dichlorofluorescein diacetate (H_2_DCFDA) to assess the presence of Reactive Oxygen Species (ROS) (Panel **C**) and the corresponding mean fluorescence index (MFI) of H_2_DCFDA (Panel **D**) in the same subgroups. Data are expressed as mean ± SD.

**Figure 5:**
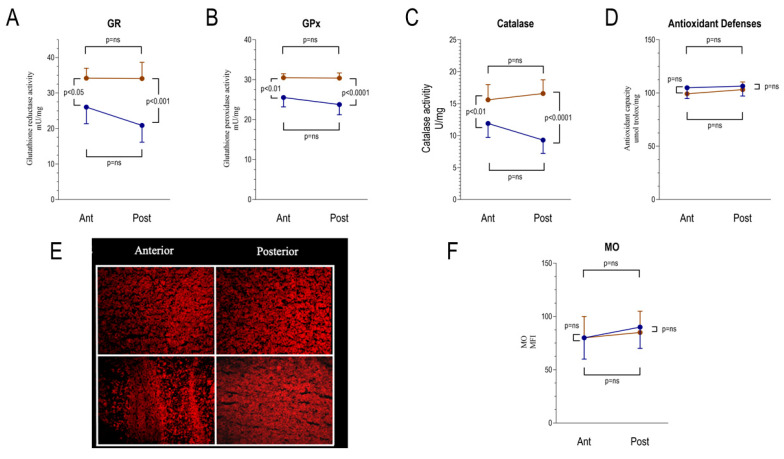
Antioxidant response to the hyperglycemia-related oxidative damage by anterior and posterior brain areas as assessed by spectrophotometry and cellular fluorescence. Panels **A**–**C** show the enzymatic activity of glutathione reductase (GR), glutathione peroxidase (GPx), and catalase (CAT) in the anterior (Ant) and posterior (Post) brain regions in control (*n* = 6 for each area, blue) and STZ mice (*n* = 6 for each area, brown). Panel **D** shows the total antioxidant capacity, evaluated as Trolox equivalent in the same subgroups. Panel **E** displays representative images of anterior and posterior brain areas stained with Mercury Orange (MO) and the corresponding mean fluorescence index (MFI, Panel **F**) in the same subgroups. Data are expressed as mean ± SD.

The decrease in ER-PPP activity of posterior brain regions exposed to chronic hyperglycemia was paralleled by enhanced oxidative damage. The decrease in ER-PPP activity of posterior brain regions exposed to chronic hyperglycemia was paralleled by the enhanced oxidative damage. In particular, the posterior brain region showed a significant increase in the superoxide anion production (Figure 4A) and in malondialdehyde (MDA) levels, a marker of lipid peroxidation (Figure 4B). A similar result was obtained when reactive oxygen species (ROS) content was estimated by H_2_DCFDA staining (Figure 4C,D). Indeed, hyperglycemia increased ROS content also in the anterior brain region, even though the enhancement was less evident in comparison to the posterior areas, where H_2_DCFDA intensity and superoxide anion production became significantly higher with respect to the anterior ones. By contrast, both the anterior and the posterior brain regions in the hyperglycemia model showed a significant increase of glutathione reductase (GR), glutathione peroxidase (GPx), and catalase (CAT) activity (Figure 5A–C), although the difference between control and STZ sample appeared more evident in the posterior region. However, despite this attempt to ameliorate the antioxidant response, the total antioxidant capacity, evaluated as Trolox equivalent (Figure 5D) and by Mercury Orange (MO) staining, remained similar in control and STZ-treated brain regions (Figure 5E,F).

### 2.4. FDG Uptake Heterogeneity Tracks the Divergent ER-PPP Antioxidant Response to the Hyperglycemia-Related Redox Damage

**Figure 6:**
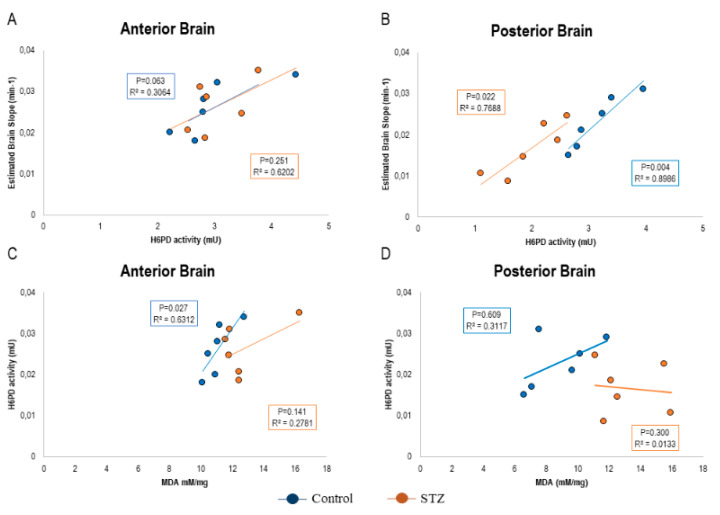
Correlation between FDG uptake, Hexose-6-phosphate dehydrogenase (H6PD) enzymatic activity, and the hyperglycemia-related redox damage at a regional level. The correlation plots between the slope of Patlak regression, H6PD enzymatic activity, and malondialdehyde (MDA) content are displayed in the anterior (Panels **A** and **C**) and posterior brain (Panels **B** and **D**) of control (blue) and STZ (brown) mice. Due to the design of the study protocol, only 12 mice (*n* = 6 controls and *n* = 6 STZ) underwent both FDG PET and biochemical analyses. R^2^ and P values of the Pearson correlations are reported in each panel.

Altogether, observed data thus suggested a selective increase in redox stress in posterior brain regions of STZ-mice that also showed a selective decrease in H6PD catalytic function. These observations suggest a possible role for the decreased ER-PPP activity both in impairing FDG uptake and enhancing the redox imbalance. We tested this hypothesis in the 12 brains that underwent biochemical analyses. In this subgroup, the avidity for the tracer (estimated by the slope of Patlak regression line) directly correlated with H6PD enzymatic activity in both anterior and posterior cortex of both control and STZ mice (Figure 6A,B). On the other hand, the link between H6PD catalytic function and MDA content was directly correlated in all subgroups but the posterior brain of STZ mice, in which the reduced enzyme function was paralleled by an increased MDA content (Figure 6C,D). 

## 3. Discussion

In the present experimental study, prolonged mild hyperglycemia elicited a heterogeneous response of brain metabolism characterized by a selective decrease of cortical FDG uptake in the posterior brain regions. At the compartmental analysis of tracer kinetics, this response was explained by a decreased FDG avidity in the posterior cortex of STZ mice, without affecting the anterior brain. This heterogeneity was not explained by the behavior of tracer entrapment mechanism since HK catalytic function was superimposable in both brain locations and independent of previous exposure to STZ. Similarly, it was not explained by the FDG-6P release machinery, since the expected [22] increase in G6Pase activity induced by sustained hyperglycemia was analogous in anterior and posterior brain regions. Finally, it was not related to any effect of STZ on the catalytic function of PFK or G6PD. Since these enzymes regulate the rate-limiting steps of glycolysis and PPP, respectively, the inhomogeneous response of FDG uptake seems to be apparently independent of the activation of pathways regulating cytosolic glucose degradation. By contrast, the expected, direct, correlation between FDG uptake and H6PD catalytic function suggests that sustained hyperglycemia was followed by a divergent activation of reticular PPP in posterior and anterior brain areas. 

On the methodological ground, the limited spatial resolution of micro-PET imaging, together with the lack of co-registered CT or MRI images, did not permit us to accurately identify whether brain FDG uptake distribution reproduces the abnormalities previously reported in some [23], though not all [24], transgenic murine AD models. Thus, the present data did not permit us to verify the degree of similarity of brain metabolic pattern in hyperglycemic and transgenic AD-like mice. Nevertheless, the different FDG uptake in anterior and posterior brain regions, observed with our rough (yet robust) analysis criterion, is coherent with several clinical studies [5,6,7,8], suggesting a possible confounding role of even moderate hyperglycemia in the clinical evaluation of AD. Ishibashi et al. [25] actually reported a heterogenous response of cortical FDG uptake to the transient increase in glucose availability induced by glucose load. Similarly, mild-fasting hyperglycemia was associated with reduced FDG uptake in the AD-related posterior cortical regions at the voxel-based quantitative analysis in at least two different studies accounting for >150 patients [5,6]. Thus, although normalization for different encephalic structures (pons and cerebellum) might partially overcome this important limitation [26], these data suggest that the specificity of brain FDG imaging might be relatively hampered in subjects with even mild increase in serum glucose level (<160 mg/dL). On the other hand, the absent interference of serum glucose level on FDG distribution indicates that the inhomogeneous brain tracer retention might parallel an inhomogeneous adaptation to prolonged hyperglycemia and its related shift in substrates’ availability.

From the pathophysiological point of view, the selective decrease in tracer uptake of posterior brain and its relative adherence with the AD-like pattern confirm the role of even moderate increases in serum glucose levels as a significant source of heterogeneity in brain FDG distribution. This response is not related to measurable alterations in cytosolic glucose degradation machinery. By contrast and in agreement with previous observations in different organs and tissues [13,14,15,16,17,18,19,20,21], it closely matches the response of H6PD catalytic function and, thus, the rate of a PPP selectively located within the ER. Since a major PPP role is the reduction of NADP to NADPH to feed the glutathione-dependent response to oxidative stress [27,28], the selective decrease in H6PD catalytic function suggests a greater vulnerability of the posterior cortex to the increased generation of ROS associated with prolonged hyperglycemia [29]. In agreement with this concept, both superoxide anion and H_2_DCFDA intensity increased selectively in the posterior brain regions. However, the increment of the oxidative stress seems to trigger the enhancement of some enzymatic activities involved in the antioxidant defenses, such as GR, GPx, and CAT. This is a physiological response since the oxidative damage depends on the unbalance between oxidative stress production and the cellular antioxidant capacity. However, this attempt to restore the balance appeared not sufficient considering the high level of lipid peroxidation and the unmutated level of total antioxidant capacity in STZ samples. This could depend on the impairment of H6PD and the relatively low NADPH level. In other words, despite the increment of GR, GPx, and CAT, lacking the NADPH, these activities are not sufficient to counteract the increment of oxidative stress. Altogether, these data thus indicate that sustained moderate hyperglycemia eventually results in significant redox stress that is more severe in the posterior cortex due to the missing response of H6PD activity, suggesting an unexpected relevance of reticular PPP in brain antioxidant response. Obviously, the present data do not elucidate whether this observation reflects a direct neuronal response or arise from the interference of activated microglia and infiltrating macrophages whose activation involves the enhancement of H6PD function [30]. Inflammatory macrophages have been reported to induce brain damage under hyperglycemic conditions in STZ-treated mice [31,32]. More importantly, hyperglycemia preferentially selectively promotes microglial activation, astrocytosis, and leukocyte recruitment in the hippocampus and, thus, in an extension of the temporal part of the cerebral cortex [33].

## 4. Materials and Methods

### 4.1. In Vivo Study Protocol 

Experiments were conducted under the Guide for the Care and Use of Laboratory Animals (Italian 26/2014 and EU 2010/63/UE directives) [34], were reviewed and approved by the Licensing and Animal Welfare Body of Ospedale Policlinico San Martino of Genoa and by the Italian Ministry of Health (Ministry authorization No. 832/2016/PR), and were performed in compliance with the “ARRIVE guidelines (Animal Research: Reporting in Vivo Experiments)”.

The study protocol included 40 6-week-old male BALB/c mice (Charles River, Italy). A subgroup (*n* = 23) received a single intraperitoneal administration of Streptozotocin at the dose of 150 mg/kg (STZ, Sigma Aldrich, St. Louis, USA), while the remaining 17 controls received saline. As reviewed by Deeds et al. [35], sensitivity to STZ is variable in the different mouse strains and is lowest in BALB/c mice. Aiming to avoid the occurrence of high glucose levels and the consequent contraindication to clinical FDG imaging, we reproduced the experimental model of Hayashi and coworkers [36], who showed the absent increase of serum glucose level for as long as 12 weeks after a single i.p. injection of STZ 150 mg/kg. To verify the occurrence of the STZ effect, 14 days later, we performed an oral glucose tolerance test (OGTT), administering a glucose load (1 g/kg) by gavage and assaying serum glucose level at 15, 30, 60, and 120 min.

### 4.2. Micro-PET Imaging Protocol and Image Processing 

**Figure 7:**
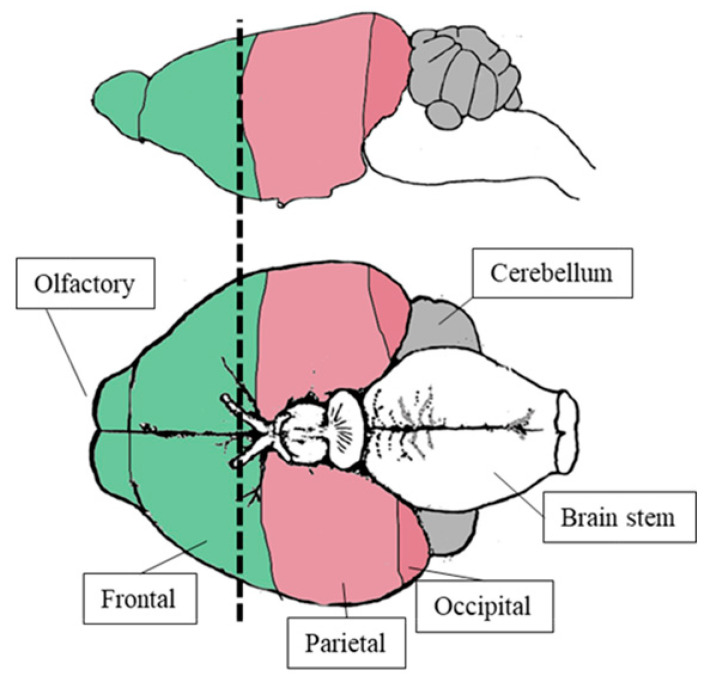
Anterior (green) and posterior (pink) brain cortex partition. As displayed, the brain was cut just in front of the optic chiasm. Consequently, the “anterior segment” mostly included frontal and olfactory cortex, while the posterior one mainly encompassed parietal–temporal and occipital lobes with the possible contamination of a small fraction of the midbrain.

Fourteen days thereafter, all mice were fasted for 12 h, weighed, and anesthetized with intraperitoneal administration of ketamine 100 mg/kg (Imalgene, Milan, Italy) and xylazine 10 mg/kg (Bio98, Italy). FDG imaging was performed as previously described [13,14,15,16,17,18,19,20,21]. Briefly, serum glucose level measurement and animals were positioned in a dedicated micro-PET system (Albira, Bruker, USA), and FDG (3–4 MBq) was injected. A 50-min list-mode acquisition was performed, binning the acquisition in the following frames: 10 × 15 s, 5 × 30 s, 2 × 150 s, 6 × 300 s, and 1 × 600 s. Images were reconstructed using the ordered subset expectation maximization method (OSEM). A volume of interest (VOI) was drawn by consensus by two expert PET readers (A.M., V.C.) in the left ventricle. Obtained data were used to plot the arterial input function as an index of FDG clearance. Parametric maps of the FDG accumulation rate were thus obtained according to the Gjedde–Patlak graphical approach [15] using commercially available software (PMOD version 3.4, PMOD Technologies Ltd., Adliswil, Switzerland). On these parametric maps, a VOI, including the anterior and posterior brain cortex (Figure 7), was performed to estimate the avidity for FDG defined by the slope of the Patlak regression line (*k*_1_ × *k*_3_)/(*k*_2_ + *k*_3_). The same VOIs were thus transferred on the last 600-s frame to estimate the corresponding average standardized uptake value (SUV) parameters.

### 4.3. Histochemical Analysis 

The day after imaging, mice were euthanized by cervical dislocation. Six randomly selected explanted brains per group were harvested and divided into anterior and posterior regions according to the same partition performed for the images’ analyses (Figure 7). Explanted brains were immediately snap-frozen in OCT, and serial cryostat sections were obtained. According to our standard procedure [16], at least three sections were stained with the sulfhydryl-reactive dye Mercury Orange (MO; Sigma–Aldrich). Aiming to confirm the specificity of MO binding to nonprotein thiols, control sections were pretreated with 100 mM N-ethylmaleimide (Sigma–Aldrich) for 10 min to block thiol groups. For ROS staining, the same number of sections obtained from the same brain samples were fixed for 10 min in acetone, incubated with 10 Μm 2′,7′-dichlorofluorescein diacetate (H_2_DCFDA; Molecular Probes) for 30 min at 37 °C, and washed in PBS. Cellular fluorescence was measured on the SP2-AOBS confocal microscope (Leica Microsystems, Mannheim, Germany). Original unadjusted and uncorrected images were processed using ImageJ software for the evaluation of fluorescence intensities with the background threshold set as previously described [37].

### 4.4. Spectrophotometric Assays 

Further, six explanted brains per group were harvested, divided into anterior and posterior regions, and immediately frozen in liquid nitrogen and stored at –80 °C. These samples were homogenized in phosphate-buffered saline (PBS) supplemented by protease inhibitors with a Potter–Elvehjem homogenizer, and enzymatic assays were performed as previously described [16]. 

Enzymatic assays were performed in a double-beam spectrophotometer (UNICAM UV2, Analytical S.n.c., Italy) [16]. HK, H6PD, and G6PD activities were assayed following the NADP reduction at 340 nm. Phosphofructokinase (PFK) activity was assayed following the NADH oxidation at 340 nm. The following assays solutions were used: (1) HK: 100 mM Tris–HCl pH 7.4 (TRIS7.4), 2 mM MgCl_2_, 200 mM glucose, 1 mM ATP, 0.5 mM NADP, 4 μg G6PD (Sigma–Aldrich); (2) H6PD: 100 mM TRIS7.4, 10 mM 2-deoxy-glucose-6 phosphate (2DG6P), 0.5 mM NADP mM; G6PD: 100 mM TRIS7.4, 10 mM glucose-6 phosphate (G6P), 0.5 mM NADP; and (3) PFK: 100 mM Tris–HCl pH 8, 2 mM MgCl_2_, 5 mM KCl, 2 mM fructose-6-phosphate, 1 mM ATP, 0.5 mM phosphoenolpyruvate (PEP), 0.2 mM NADH, 4 μg pyruvate kinase (PK)/lactate dehydrogenase (LDH) (Sigma–Aldrich). Glutathione reductase (GR) and glutathione peroxidase (GPx) activity in brain homogenates were evaluated spectrophotometrically at 405 and 450 nm, respectively, using a GR assay kit (Abcam: ab83461) and GPx assay kit (Abcam: ab102530), following the manufacturer’s instructions. Catalase activity was assayed in PBS in the presence of 5 mM hydrogen peroxide, following the absorbance decrease at 240 nm [38]. The general antioxidant defenses were evaluated by the Antioxidant Assay Kit (Sigma-Aldrich: MAK334-1KT), following the manufacturer’s instructions. The production of superoxide anion was measured by mean of the difference between total and 300 U SOD-inhibitable cytochrome c reduction at 550 nm, as previously described [39]. 

Glucose in the medium was assayed following the reduction of NADP at 340 nm. The following solutions were used: glucose: 100 mM TRIS7.4, 2 mM MgCl_2_, 2 mM ATP, 4 μg HK/G6PD (Sigma–Aldrich). G6Pase activity on G6P was measured at 660 nm, following the inorganic phosphate production [16], using 100 μg brain proteins. The assay solution contained 60 mM Tris-maleic acid pH 6.5 and 40 mM G6P. Following incubation at 37 °C for 15 min, the reaction was blocked with 2% trichloroacetic. Samples were centrifuged at 14,000 rpm for 2 min, and the supernatant was added with 350 mM H_2_SO_4_, 350 mM ammonium molybdate, and 10 μL of 2.5 mg/mL Fiske’s reactive (710 μL of final volume). 

Aiming to assess lipid peroxidation, malondialdehyde (MDA) levels were evaluated, by the thiobarbituric acid reactive substances (TBARS) assay, with minor modifications [16].

### 4.5. Western Blot Analysis

Expression of the enzymes involved in glucose metabolism and in cytosolic and ER PPP was determined by Western blot, using standard procedures. Anterior and posterior brain regions were homogenized in PBS in the presence of a protease inhibitor cocktail for mammalian cells, and total protein was measured by Bradford assay. After SDS-PAGE, performed according to the standard method on 10% precast gels (BioRad), proteins were transferred to a nitrocellulose membrane. The membrane was blocked for 1 h with Tris Buffered Saline (TBS) plus 0.15% Tween 20 (TBSt) containing 5% nonfat dry milk and incubated overnight at 4 °C with the following rabbit polyclonal antibodies: anti-HK II (1:1000, Cell Signalling #2867), anti-G6Pase (1:1000, Abcam, ab93857), anti-PFKP (1:200, Cell Signalling #8164), anti-G6PD (1:1000, Abcam, ab210702), anti_H6PD (1:1000, Abcam, ab170895), or anti-actin (1:10,000, Thermo-Fisher MA5-11869). After washing with TBSt, the membrane was incubated with an anti-rabbit IgG antibody conjugated with horseradish peroxidase (HRP) (BioRad) and developed with Clarity Western ECL Substrate (BioRad). Bands were detected and analyzed for density using an enhanced chemiluminescence system (Alliance 6.7 WL 20 M, UVITEC, Cambridge, UK) and UV1D software (UVITEC). Bands of interest were normalized for actin levels in the same membrane. 

### 4.6. Statistical Analysis 

Data are presented as mean ± standard deviation. For comparisons, the Student *t*-test for paired or unpaired data was applied, as appropriate. Synergism was tested using a factorial experimental design in which the interaction factor was assessed using the univariate analysis of the general linear regression model, as described by Slinker et al. [40]. Linear regression analyses were performed using the Pearson correlation coefficient. Statistical significance was considered for *p* < 0.05. Analyses were performed using the SPSS software package, 20.0.0 release (IBM, Armonk, NY).

## Supplementary Materials

Supplementary materials can be found at https://www.mdpi.com/1422-0067/21/21/8154/s1. Figure S1: Western blot results mirror the observed differences in enzymatic activities.
