# Supplementary figures and images for: 18F-Fluorodeoxyglucose Positron Emission Tomography Tracks the Heterogeneous Brain Susceptibility to the Hyperglycemia-Related Redox Stress
Source: Int J Mol Sci. 2020 Oct 31;21(21):8154. doi: 10.3390/ijms21218154 (PMC7672601; doi:10.3390/ijms21218154)

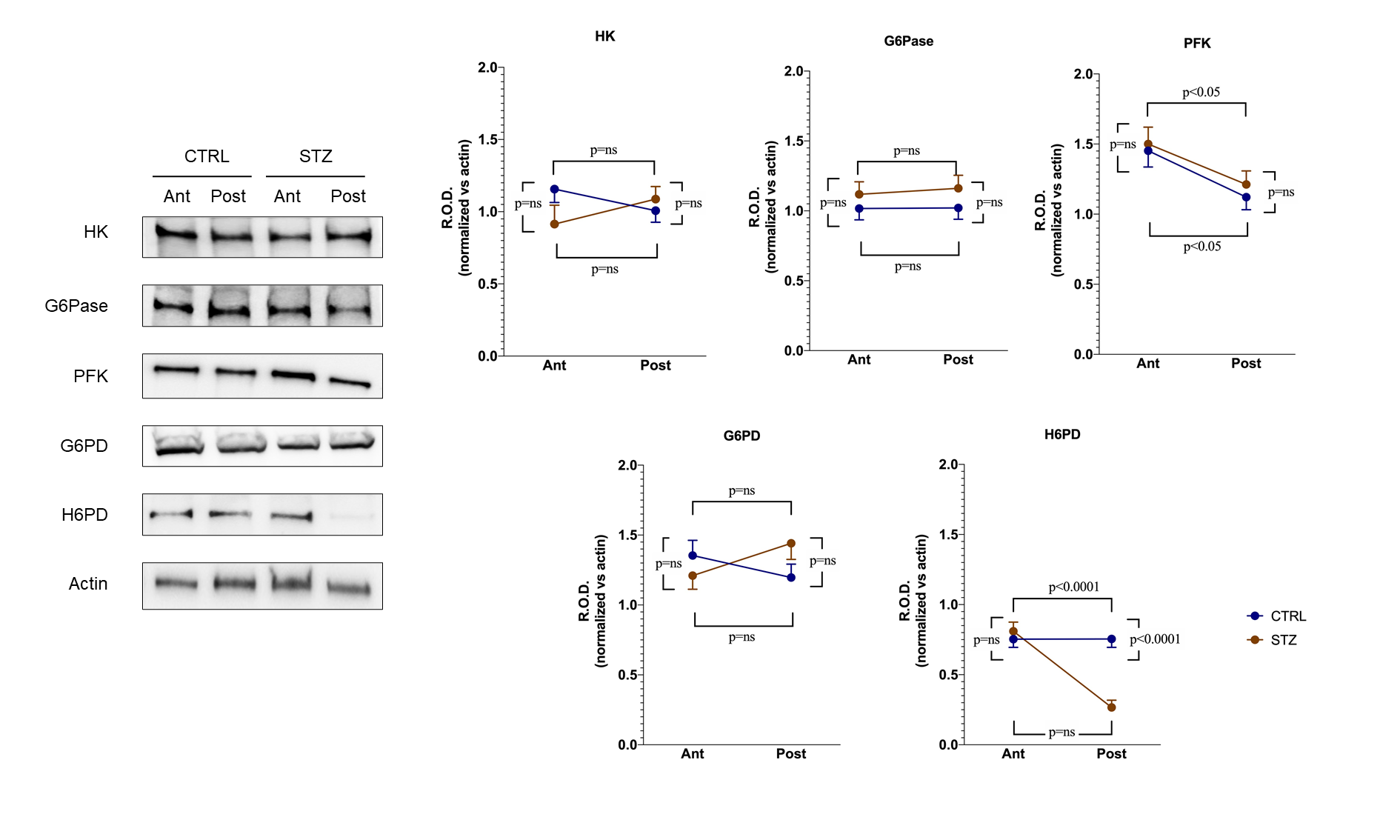

Supplement: Supplementary file 1 [file ijms-21-08154-s001.zip › ijms-951981-supplementary.tif]
